# Biographical Feature: Bernhard Fleckenstein

**DOI:** 10.1128/JVI.00896-21

**Published:** 2021-08-10

**Authors:** Andrea K. Thoma-Kress, Ronald C. Desrosiers, Michaela U. Gack, Jae U. Jung, Klaus Überla, Frank Kirchhoff

**Affiliations:** a Institute of Clinical and Molecular Virology, Friedrich-Alexander Universität Erlangen-Nürnberg (FAU), Erlangen, Germany; b Department of Pathology, Miller School of Medicine, University of Miami, Miami, Florida, USA; c Florida Research and Innovation Center, Cleveland Clinic, Port Saint Lucie, Florida, USA; d Department of Cancer Biology and Global Center for Pathogens Research and Human Health, Lerner Research Institute, Cleveland Clinic, Cleveland, Ohio, USA; e Institute of Molecular Virology, Ulm University Medical Centre, Ulm, Germany; University of California, Irvine

**Keywords:** Bernhard Fleckenstein, obituary, human herpesviruses, mentoring, retroviruses

## TEXT

Bernhard Fleckenstein, Professor Emeritus and former Chair of the Institute of Clinical and Molecular Virology at the Friedrich-Alexander-University Erlangen-Nuremberg (FAU), Erlangen, Germany, passed away on 4 May 2021. Virology has lost a remarkable personality who dedicated his scientific and academic career to oncogenic herpesviruses, the promotion of the field of virology, and the training and support of young scientists. His colleagues, fellows, and friends mourn a sympathetic, humorous, energetic, and creative scientist who was an appreciated supervisor and colleague, as well as a wonderful mentor and friend. To remember Bernhard Fleckenstein and his accomplishments in virology research, mentorship, and academia, this biographical feature not only will present the main stages of his academic career and his major scientific achievements but also aims to provide insights into his admirable personality.

### The early years.

Bernhard Fleckenstein was born in Würzburg, Germany, on 10 August 1944 as the first son of the physiologist Albrecht Fleckenstein and his wife Ilse Fleckenstein. He grew up in Würzburg, Heidelberg, and Freiburg, Germany. From 1963 to 1969, he studied Medicine at the University of Freiburg and University of Vienna. After passing his state examinations in Medicine in Freiburg in 1969, he worked as a medical assistant in hospitals at Karlsruhe and Lübeck, Germany. In 1970, he was awarded his M.D. degree in the field of physiology at the University of Freiburg. His thesis focused on Ca^2+^ antagonists and their impact on inhibiting contractility of the myocard. After obtaining his M.D. degree, he worked as a scientific assistant at the Institute for Hygiene and Microbiology at the University of Göttingen, Germany (Chair, Prof. Reiner Thomssen). During this time, he started to develop his enthusiasm for research on viruses and especially tumor viruses. In 1972, he moved to Erlangen, Germany, and worked on his habilitation at the Institute of Clinical Virology at the FAU directed by Professor Harald zur Hausen, a renowned expert on papillomaviruses and later a Nobel laureate. In 1975, Bernhard Fleckenstein finished his habilitation thesis, which focused on oncogenesis by herpesvirus saimiri (HVS) in primates, a model for herpesvirus-induced tumor development in humans. He stayed in Erlangen as “privatdozent,” which is comparable to assistant professor, until 1976.

### Moving to the United States.

From 1976 to 1978, Bernhard Fleckenstein held the position of Associate Professor of Microbiology and Molecular Genetics at Harvard Medical School (HMS), Boston, MA, and head of the Department of Microbiology at the Primate Research Center at Harvard University. He was closely connected with this institution not only scientifically but also on a personal level for more than 4 decades. Coauthor R.C.D. was hired into his first faculty appointment by Bernhard Fleckenstein during this period. They not only published 10 papers together over their careers but also hiked, traveled, enjoyed wine, and visited each other on both sides of the ocean whenever the opportunity arose. Bernhard Fleckenstein’s link with HMS and its primate research center was instrumental in the establishment of the highly successful joint graduate student training program GRK1071 between FAU and HMS from 2005 to 2013.

### Back to Germany: expansion of the Institute of Virology in Erlangen.

At the age of 34 years, Bernhard Fleckenstein was appointed Full Professor and Chair of Virology at the FAU, Erlangen, Germany. For more than 36 years (1978 to 2015), he was Director of the Institute of Clinical and Molecular Virology in Erlangen. He not only established a broad research program on herpesviruses, papillomaviruses, and retroviruses but also expanded the virological diagnostics section. The latter also benefitted from his medical specialization in microbiology and infection epidemiology, which he finished in 1981. Bernhard Fleckenstein was a very dedicated and generous mentor. He supported numerous junior researchers in virology, both in Germany and the United States, including all authors of the present article. Many of his trainees and “descendants” have been appointed Full Professors, Chairs, Deans, and Institute Directors at prestigious national or international institutions. For more than 16 years (1996 to 2012), he was speaker of the National Reference Center for Retroviruses (NRZ) in Erlangen, the German surveillance center for retroviruses, which is appointed by the Federal Ministry of Health and the Robert Koch Institute (Berlin) every 3 years. Bernhard Fleckenstein was loyal to the Institute of Virology at FAU; he refused several calls as Chair for other institutions, including Chair of Virology at the Universities of Freiburg (1987) and Munich (1994), and as a scientific member and Chairman of the Foundation Board of the German Cancer Research Center (DKFZ) in Heidelberg (2003). Instead, he expanded both the institute and the diagnostics section of the Institute of Virology in Erlangen not only scientifically but also structurally to a national and international renowned center for clinical and molecular virology which employs more than 100 scientists and technicians.

### Scientific achievements.

Bernhard Fleckenstein had a great passion for oncogenic herpesviruses and other tumor viruses (1, 2). His research was focused on the oncogenic rhadinoviruses (γ2 herpesviruses) and their impact on the development of lymphomas. He started his scientific career in molecular virology focusing on herpesvirus saimiri and ateles (HVS and HVA, respectively), rhadinoviruses that naturally infect nonhuman primates. His first publication demonstrated that HVS induces malignant lymphoma in a New World monkey and can be reisolated from a tumorous lymph node of the infected animal after short cultivation *in vitro* (3). Later, he published that isolated rhadinoviral DNA is tumorigenic *in vivo* (4). His group deciphered the genome structure of rhadinoviruses and found that rhadinoviruses from nonhuman primates are able to transform human T cells *in vitro* (see details in Selected Bibliography). The discovery of rhadinoviral oncoproteins improved our understanding of signaling pathways contributing to T cell transformation. In his pioneering work to develop rhadinoviral vectors, Bernhard Fleckenstein also contributed to research deciphering T cell transformation induced by human T cell leukemia virus type 1 (HTLV-1), the only human oncogenic retrovirus. Later, he also worked on Kaposi’s sarcoma-associated herpesvirus (KSHV), another representative of the rhadinoviruses which infects humans and can cause malignancies in immunosuppressed individuals, such as AIDS patients (5). He discovered immune-modulating proteins of KSHV, mapped KSHV structural proteins, and contributed to the discovery of the KSHV receptor.

In addition to oncogenic herpesviruses, Bernhard Fleckenstein had a particular research interest in human cytomegalovirus (HCMV), which can cause severe diseases in immunosuppressed patients or during prenatal infection. He cloned the entire HCMV genome of 235 kb and mapped the structural and regulatory viral proteins. In collaboration with Walter Schaffner from the University of Zurich, Switzerland, Bernhard Fleckenstein discovered the immediate early enhancer of HCMV in 1985 (6). This was a major discovery, as this enhancer is still used worldwide to express eukaryotic genes or monoclonal antibodies in cell culture models or transgenic animals and for the production of therapeutic proteins for use in humans (7–9).

In addition to these achievements, Bernhard Fleckenstein had a major research interest in T cell transformation and worked on this topic even after his retirement in 2015. He collaborated with different laboratories working on inherited diseases, which led to numerous publications in respected journals. In these projects, Bernhard Fleckenstein expanded primary human CD4^+^ T cells from patients with rare gene defects by using his rhadinoviral vectors, which allowed for a better understanding of the signaling pathways underlying certain rare diseases. Moreover, Bernhard Fleckenstein was a strong supporter of research on HTLV-1 at the Institute of Virology in Erlangen.

Bernhard Fleckenstein’s scientific productivity is illustrated by his more than 250 published articles, including more than 160 original research articles (see a brief summary below in Selected Bibliography). His studies were published in leading scientific journals, including Nature, Cell, Proceedings of the National Academy of Sciences (PNAS), Journal of Experimental Medicine, and Journal of Virology. In the latter, he also served as a reviewer and member of the Editorial Board.

### Commitment for science, virology, and academia.

Bernhard Fleckenstein was active in many national and international boards of science and in numerous committees of the FAU working on academic self-administration. He was a member of the “Akademie der Wissenschaften und der Literatur zu Mainz” and of the German national academy of Sciences “Leopoldina.” Moreover, he was a scientific advisor of the “Wilhelm-Sander Foundation” for more than 30 years and for a long period of time even as a chairman. At FAU Erlangen, he was Dean of the Faculty of Medicine from 1997 to 2001 and from 2005 to 2008. During this time as well as later, he helped to pave the path for performance-oriented research funding at the University Hospital Erlangen. Bernhard Fleckenstein also served as Chair of the Foundation Council of “Forschungsstiftung Medizin” at the University Hospital Erlangen.

Training and mentoring young scientists was very important to Bernhard Fleckenstein. The number of undergraduate and graduate students he supervised during his career is essentially uncountable. He was always more than willing to serve as a reviewer in thesis committees and enjoyed educating students. His commitment to educate young scientists covered all levels of a scientific career. In the 1990s, he recruited several group leaders to strengthen HIV research in Erlangen, and among them were two of the coauthors, namely, K.Ü. and F.K. This helped Bernhard Fleckenstein to extend the institute’s broad expertise on herpesviruses to retroviruses and to establish the collaborative research center SFB 466 “Lymphoproliferation and viral immunodeficiency” (1996 to 2007). Later, he was chairman of the research training grant GRK1071 “Viruses of the immune system”; both initiatives were funded by the German Research Foundation (DFG), and the latter was performed in close collaboration with H.M.S. and R.C.D.

Bernhard Fleckenstein also strongly shaped the field of virology in Germany and Europe. He was founding president of the German Society of Virology (1990 to 1996), the professional society of German-speaking virologists, which has currently more than 1,000 members. After 1996, he stayed closely connected with the society as member of the advisory board. Furthermore, Bernhard Fleckenstein was a founding member and “Secretary General” of the European Society for Virology (2009). Together with Otto Haller (Freiburg), he hosted the Third European Congress of Virology of the European Society for Virology in Nuremberg, Germany, in 2007. Bernhard Fleckenstein’s passion for virology is also reflected by his voluntary memberships in the German “Standing Committee on Vaccination” of the Robert Koch Institute (from 1998 to 2004) and his participation in the “National commission to eradicate poliomyelitis” (from 2003 to 2010).

### Awards and honors.

Bernhard Fleckenstein received numerous national and international awards and honors, including the Max Planck Prize (1991; together with R.C.D.), the Aronson Prize (Berlin, 1991), and the Ludwig Aschoff Prize of the University of Freiburg (2004). Moreover, Bernhard Fleckenstein was honored with the Cross of the Order of Merit of the Federal Republic of Germany (2003) and the Bavarian Order of Merit (2006). While he was Director of the Institute of Virology, Alexander von Humboldt prizes were awarded to internationally renowned scientists, including Mark Stinski (1989), Bryan Cullen (1993), Matija Peterlin (1995), Ellen Fanning (2010), and Indrikis Muiznieks (2012), which allowed them to spend time researching at the Institute of Virology in Erlangen.

### Personality and personal life.

Our colleague, mentor, and friend Bernhard Fleckenstein was not only an insightful and far-sighted scientist with broad perspective but also a very generous person. His strong solidarity with virological research and his altruistic support of junior researchers is reflected by the establishment of the “Bernhard und Ingrid Fleckenstein-Stiftung,” a foundation that supports young scientists with a thesis prize, which is awarded at the annual meeting of the German Society for Virology. Quite clearly, Bernhard was an encouraging and supportive mentor who helped ensure that his fellows would have successful careers as independent scientists. In fact, even after his retirement, you could always call him to discuss a manuscript as well as other scientific or personal issues. Not only was an excellent scientific environment important to him but also a collegial and supportive working atmosphere as well. He took pride in supporting and funding social events like Christmas parties or leisure skiing activities. Bernhard Fleckenstein dedicated his personal life to his wife Ingrid, his children, and his family. During the last years, he was always fascinated by and proud of the fast development of his grandchildren and greatly enjoyed being a grandfather. In his spare time, he loved to go hiking, especially in the mountains. It is incredible that he knew not only geographical facts but also historical anecdotes about nearly every mountain or hill. After his retirement, he spent his time next to his home village in the Franconian Switzerland, an amazing region in the northern part of Bavaria; also in the Alps in Ellmau, Austria; and in the south of France. Despite being physically apart, he was still well connected to his colleagues, and it was not uncommon to phone with him late at night to discuss recent issues.

We remember Bernhard as a greatly supportive and generous mentor who encouraged his trainees and fellows to aim high. His general motto was “It doesn’t cost you anything.” Despite his numerous obligations, he always found time to give advice and to help. Noteworthily, he was a straightforward, practical person. One common question he asked trainees about new projects was: “What might the title of of your first manuscript on this topic be?” He was very concise and also taught his students and fellows to focus on the central theme of their research topics. Finally, Bernhard was well known for his persistence and his enthusiasm for new discoveries. His friends and colleagues greatly appreciated his support and generosity. Bernhard Fleckenstein will be greatly missed and be kept in our memories. We express our sincere condolences to his family.

### Selected bibliography.

The following sections include lists of study topics and specific articles in which Bernhard was involved.

### (i) Work on rhadinoviruses infecting nonhuman primates.

Genome structure of rhadinoviruses and development of rhadinoviral expression vectors (10–12). Discovery of *in vitro* transformation of human T cells by rhadinoviruses of nonhuman primates. Discovery of rhadinoviral oncoproteins. Insights into cell signaling during T cell transformation (13–23). Use of rhadinoviruses for T cell expansion to elucidate signaling in inherited disorders (24–27).

### (ii) Work on Kaposi’s sarcoma-associated herpesvirus (KSHV).

Discovery of immune-modulating proteins of KSHV and mapping of KSHV structural proteins (28–30). Contribution to KSHV receptor identification (31, 32).

### (iii) Work on human cytomegalovirus (HCMV).

Cloning of the HCMV genome and mapping of relevant structural and regulatory proteins (33–38). Identification and characterization of the major immediate early enhancer of HCMV and analysis of HCMV gene regulation (6, 39–42).

### (iv) Work on retroviruses (HIV and HTLV-1).

Work on HIV Rev (43, 44). Interference between HIV and Hepatitis G virus (45, 46). *In vitro* transformation of T cells by the Tax oncoprotein of HTLV-1. Demonstration that HTLV-1 Rex binds Rex-response element. Impact of Tax on T cell signaling (47–50).

### (v) Qualification theses.

These include references 51 and 52.
